# The Indicators of Clinical and Subclinical Mastitis in Equine Milk

**DOI:** 10.3390/ani12040440

**Published:** 2022-02-11

**Authors:** Dominika Domańska, Michał Trela, Bartosz Pawliński, Bartłomiej Podeszewski, Małgorzata Domino

**Affiliations:** Department of Large Animal Diseases and Clinic, Institute of Veterinary Medicine, Warsaw University of Life Sciences, 02-787 Warsaw, Poland; dominika_domanska@sggw.edu.pl (D.D.); michal_trela@sggw.edu.pl (M.T.); bartosz_pawlinski@sggw.edu.pl (B.P.); bartlomiej_podeszewski@sggw.edu.pl (B.P.)

**Keywords:** lactation, mare, electrical conductivity, somatic cell count, subclinical mastitis

## Abstract

**Simple Summary:**

Mastitis, the inflammation of the mammary gland, is a major problem in a mare’s perinatal period, negatively affecting both the health of the mare and newborn foal and the quality of milk produced on dairy equine farms. The detection of mastitis is therefore one of the important goals in the equine breeding and dairy industry. This study aimed to determine the somatic cell count (SCC), the percentage of the immune cells, the electrical conductivity (ECM), and bacteriological index (BII) in milk collected from mares with (CM) and without (NCM) clinical symptoms of mastitis. The increase in examined indicators is suspected to be a subclinical mastitis indicator, therefore the study aimed to separate two subgroups, mares with (SM) and without (NSM) subclinical symptoms of mastitis. In milk from NCM mares the values of SCC, immune cells, and ECM increased immediately after birth and weaning, whereas during the rest of the lactation period their values were at a low level. Similarly, in milk from CM mares, the values of examined indicators were high and comparable to the weaning time. An increase in the level of examined indicators may become an early indication of subclinical mastitis.

**Abstract:**

The somatic cell count in milk (SCC) and electrical conductivity of milk (ECM) are indicators of the health status of the mammary gland. Among somatic cells, mainly polymorphonuclear neutrophils (PMN), macrophages (MAC), and lymphocytes (LYM) are rated. This study aimed to determine the SCC, PMN, MAC, LYM, ECM, and bacteriological index (BII) in milk collected from mares with (CM) and without (NCM) clinical symptoms of mastitis concerning mares with (SM) and without (NSM) subclinical mastitis. Milk samples were collected from 27 mares divided into NCM (*n* = 12) and CM (*n* = 15) groups. In samples, SCC quantification, cytological examinations, ECM measurement, and bacteriological examination were performed. In NCM mares, the values of SCC, PMN, MAC, LYM, and ECM were higher in initial than in consecutive examined days after birth until weaning. After weaning the proportion of SCC, PMN, MAC, LYM, ECM, and BII increased and did not differ with the average values in CM mares. These equine milk indicators may reflect an early symptom of subclinical mastitis and in the future may be used in the early detection of mastitis or as a tool of assessment of the health status of the mammary gland in the dairy equine farm.

## 1. Introduction

Nowadays, the equine dairy industry is becoming an increasingly important part of the whole equine industry given its potential health-promoting impact in modern and future society [1,2,3,4,5]. In Europe, the dairy equine industry is thriving in France where it was started with the animal diversity preservation project [6], and it is developing little by little in many areas of the world including Italy, Greece, Germany, Mongolia, Kazakhstan, Kirgizstan, and China [7,8,9]. The interest toward equine milk and derivatives results from the compositional peculiarities of equine milk and their potential health-promoting properties, which have found applications in both human food [5,7,10,11] and non-food sectors [12]. In the food sector, the use of equine milk supports nutrition and treatment of children with allergies to bovine milk protein [7,13], children with multiple food allergies [10], and children with food protein-induced enterocolitis [11]. This is in addition to, but not as a substitute for the currently recommended preparations obtained exclusively from cow milk and preparations containing an isolated fraction of soy protein enriched with methionine [14]. Equine milk is used also in the nutrition of adults with immunocompromised or debilitated systems [7,13]. In the non-food sector, equine milk works well as a good ingredient in cosmetic products [12]. Such a specific utilization of equine milk is determined by its beneficial content of water, fat, and allergens including lactose [15], which is much closer to humans than to bovine milk [5,15]. Moreover, the presence of many bioactive and functional compounds, i.e., metabolites, enzymes, hormones, trophic, and protective factors [16,17], including a high content of lysozyme and lactoferrin [8,16,18], additionally supports the beneficial properties of equine milk. These milk components, beyond their nutritive value, exert health-promoting properties including antimicrobial, antihypertensive, antioxidant, antithrombotic, immunomodulatory, and antiproliferative activities [2,3,4]. However, it should be kept in mind that equine milk components are mainly affected by the mare’s nutrition, length of lactation, and health status of the mammary gland [5]. The health status of the mammary gland is a crucial factor affecting milk production, quality, and composition in most dairy farmed animals [19,20,21,22].

Mastitis, mammary gland inflammation, is rarely observed in dairy equine farms [23,24,25], whereas its occurrence is suspected more often concerning subclinical states [26,27]. Among the main causes of mastitis in dairy equine farms, injuries or improper milking procedures reported for more intensive farming systems are mentioned [5,24]. However, the occurrence of predilection periods for mastitis and the possibility of monitoring mare mastitis is still insufficiently investigated. In dairy equine farms, the somatic cell count (SCC) is used as an indicator of the mammary gland’s health status. When SCC is reported to be above 50,000 cells per mL of milk, the inflammatory process in the mammary gland is suspected [23,24,25], and when clinical symptoms also occur, mastitis is confirmed [23,24,25,26,27,28,29]. Based on the clinical presentation, mastitis can be classified as acute or chronic, and clinical or subclinical [30]. From a layman’s point of view, mares with acute mastitis present local clinical symptoms for less than 7 days, and the resolution of clinical symptoms occurred within 7 days after treatment initiation. Conversely, mares with chronic mastitis present local clinical symptoms for more than 7 days, and the clinical symptoms may not resolve within 7 days after treatment initiation [26,30]. Both classes represented clinical mastitis, where the presence of the clinical symptoms is an inclusion criterion [30]. Whereas subclinical mastitis represented those cases without local clinical symptoms [26], the milk production and the quality of milk decreases with the increase of SCC [28]. Unlike in cows, specific cut-offs [26] and the predilection period for the occurrence of subclinical mastitis have yet not been determined for these classifications in mares [28,29]. Therefore, any effort is required to shed new light on the classification, recognition, and population prevalence of mammary gland inflammation in mares.

In addition to clinical and bacteriological examinations, as well as determination of SCC, the electrical conductivity of milk (ECM), and acute-phase protein concentration should be taken into account in the case of the diagnosis of mastitis in mares, especially subclinical mastitis [29,31,32]. Most publications on mastitis in mares focus on bacteriological diagnostics and treatment [26,27,33,34,35], therefore this study is designed to fill the gap by demonstrating the application of cytological evaluation and electrical conductivity determination of equine milk samples as potential indicators of inflammation of the mammary gland in mares. As in dairy farmed animals, increased ECM and SCC is a useful indicator of inflammation in the mammary gland [36,37]. The relation between ECM and SCC in the healthy and inflamed mammary gland in mares has not been thoroughly investigated. We hypothesized the evaluation of ECM and SCC in equine milk may become a useful indicator of subclinical and clinical mastitis in mares. Therefore, this study aimed to determine the ECM and SCC, including the percentage of immune cell populations in the healthy and inflamed mammary gland in mares, and compare them to find co-occurrences.

## 2. Materials and Methods

### 2.1. Animals

**Figure 1:**
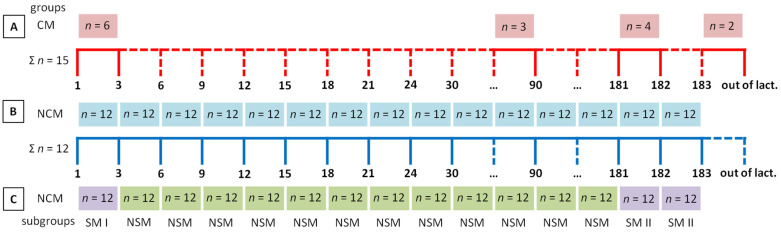
A designee of the study including the number of samples (*n*), days of lactation (1, 3, 6, 9, 12, 15, 18, 21, 24, 30, 90, 181, 182, 183), days out of lactation (out of lact.), a sum of mares in the group (Ʃ*n*), group names (CM, group with clinical symptoms of mastitis; NCM, group with no clinical symptoms of mastitis), subgroup names (SM I, SM II, group with subclinical symptoms of mastitis; NSM, group with no subclinical symptoms of mastitis). Sampling in the group with clinical symptoms of mastitis (**A**). Sampling in the group with no clinical symptoms of mastitis (**B**). Dividing the group with no clinical symptoms of mastitis into two subgroups, the first with subclinical symptoms of mastitis and the second with no subclinical symptoms of mastitis based on the results of the studied measurements (**C**).

The study was carried out on mares housed in the Horses Stable Krasne (Krasne, Poland). The total number of twenty-seven thoroughbred mares (*n* = 27) (aged 3 to 16 years, body weights 400 to 700 kg) included two groups: fifteen mares in a group with clinical symptoms of mastitis (CM, *n* = 15) and twelve other mares in a group with no clinical symptoms of mastitis (NCM, *n* = 12). Mares included in the CM group were not a part of the NCM group (Figure 1).

The NCM group’s inclusion criterion was the lack of clinical symptoms of inflammation of the mammary gland throughout the sampling period. The CM group’s inclusion criterion was the presence of the clinical symptoms of inflammation of the mammary gland throughout the sampling period. The clinical symptoms of inflammation of the mammary gland were assessed during a clinical examination conducted following the standard protocol [30]. The inflammation of the mammary gland was recognized when mammary gland pain, local swelling or heat in the affected gland, gland asymmetry, gland firmness, ventral oedema with or without concomitant lower limb oedema, a congested mammary vein, rejection of the foal, and abnormal mammary gland secretions were observed [30,33]. The clinical symptoms occurred in mares in the mastitis group at different times of lactation as follows: postpartum (*n* = 6; 1st, 1st, 2nd, 2nd, 3rd, and 3rd day of lactation), during lactation (*n* = 3; 76th, 90th, and 90th day of lactation), after weaning (*n* = 4; 181st, 181st, 181st, and 182nd day of lactation), and in the non-lactating period (*n* = 2; out of lactation).

All mares were housed in the Horses Stable Krasne under the same environmental conditions. All mares received an individually calculated ration of hay, oats, and concentrate according to their nutritional requirements. The ratio of feed was distributed over three feedings per day. A mineral salt block and freshwater were constantly available.

### 2.2. Sample Collection

Samples of milk and inflammatory secretions from the mammary gland were collected according to the standard protocol [35]. The samples were collected into sterile tubes containing no preservatives, after cleaning and disinfecting the teats (with particular attention to the top of the teat). The disinfection process started from the teat situated farther, and the collection of the material was started from the teat located closer. After collection, the samples were cooled to 4 °C and thereafter transported to the laboratory of the Department of Large Animal Diseases and Clinic, Institute of Veterinary Medicine, Warsaw University of Life Sciences. The samples were tested within two hours of collecting the material.

The material in the CM group was collected once, after a recognition of the symptoms of the inflammation of the mammary gland, before applying treatment and drying up the mares. The material in the CM group was collected from one mare each time: in the postpartum period (*n* = 6; after normal parturition: *n* = 4; 1st, 1st, 2nd, and 3rd day of lactation, and after cesarean section: *n* = 2; 2nd and 3rd day of lactation), during lactation (*n* = 3; 76th, 90th, and 90th days of lactation), after weaning (*n* = 4; 181st, 181st, 181st, and 182nd days of lactation), and in the non-lactating period (*n* = 2; out of lactation) (Figure 1A).

The material in the NCM group was collected fourteen times from twelve mares each time: on 1st, 3rd, 6th, 9th, 12th, 15th, 18th, 21st, 24th, 30th, and 90th day after foaling, and in the first three days after weaning (181st, 182nd, and 183rd day after foaling) (Figure 1B).

### 2.3. Examination of Milk and Inflammatory Secretions from the Mammary Gland

The quantification of somatic cells was performed with a Somacount TM 150 counter (Bentley Instruments. Inc., Chaska, MN, USA). Samples of milk and inflammatory secretions (0.10 mL) were measured after the temperature spontaneously returned from 4 °C to room temperature and gently mixed. A fluorescent dye was added to the milk samples, which stains the DNA of somatic cells. The somatic cells stained and excited by the laser beam were counted utilizing software based on the image recorded with the CCD matrix of the device. The SCC result was expressed as the number of cells in 1 mL of milk (×10^3^/mL).

Cytological examination of milk and inflammatory secretions was carried out according to standard protocol [38,39]. Samples of milk and inflammatory secretions (0.01 mL) were spread on a plate and left to dry at room temperature (smearing). The smear was then dipped in xylene and ethanol, 5 min in each of the reagents, and then the slide was placed for 90 s in a cuvette with dye. After the staining was finished, the samples were analyzed and assessed microscopically (Olympus BX53, Olympus Polska Sp. z o.o., Warsaw, Poland) at 100× magnification with the use of immersion. For each smear, 20 visual fields were assessed. The somatic cells were differentiated into polymorphonuclear neutrophils (PMN), macrophages (MAC), and lymphocytes (LYM) [40]. The cytological result was expressed as the percentage of PMN, MAC, and LYM of all SCCs observed in the smear (%).

The tests of ECM were conducted using the Mastitron (Fa. Milku, Bovenden, Lenglern, Germany). Samples of milk and inflammatory secretions (2.0 mL) were thawed before measuring. The ECM result was expressed as millisiemens per centimeter (mS/cm) according to the equine protocol [41,42].

Bacteriological examination of milk was carried out according to standard protocol [35]. Samples of milk and inflammatory secretions (0.01 mL) were plated on McConkey, Edwards, and Sabouroud blood agar. When any growth was noted after standard incubation, identification of the isolated bacteria was performed on the basis of API tests (bioMérieux, Durham, NC, USA). The milk sample was annotated as bacteriological positive (1) when bacterial growth on plates was observed and identified. The milk sample was annotated as bacteriological negative (0) when no growth on plates was observed. The positive and negative results were presented as a data series for each day of material sampling, and the Bacterial Infection Index (BII) was calculated as a mean value of each data series independently. The values of BII ranged from 0.0 to 1.0.

### 2.4. Statistical Analysis

All statistical analyses were performed using GraphPad Prism6 software (GraphPad Software Inc., San Diego, CA, USA), where the significance level was established as *p* < 0.05. The data distributions of the data series of SCC, PMN, MAC, LYM, ECM, and BII were tested independently using the univariate Kolmogorov-Smirnov test. Data series were tested independently for each of the fourteen samples of the NCM group and one sample of the CM group.

Firstly, data series of SCC, PMN, MAC, LYM, ECM, and BII were compared between the left and right half of the mammary gland using a paired *t*-test for Gaussian data and the Wilcoxon matched-pairs signed-rank test for non-Gaussian data. As no differences were found between two halves for each of the examined indicators, the left and right half’s data sets were pulled and used as entire data sets in the further comparisons.

Secondly, fourteen samples of the NCM group were compared between the days of milk sampling as paired data, independently for each parameter (SCC, PMN, MAC, LYM, ECM, and BII). The comparisons were assessed by the Friedman test, followed by Dunn’s multiple comparisons test, due to the non-Gaussian distribution of at least one data series in each data being compared. When data series differed significantly (*p* < 0.05) between the days of sampling independently for SCC, PMN, MAC, LYM, ECM, and BII, the significantly higher level of milk indicator became a criterion for the recognition of subclinical mastitis. Based on the results, the NCM group was divided into two subgroups representing the same number of mares (*n* = 12) but different sampling days: the group with no subclinical symptoms of mastitis (NSM, *n* = 12) and the group with subclinical symptoms of mastitis (SM, *n* = 12) (Figure 1C). The NSM group’s inclusion criterion was the lack of the clinical symptoms of inflammation of the mammary gland and the lack of significantly higher levels of SCC, PMN, MAC, LYM, ECM, or BII in milk samples. The SM group’s inclusion criterion was the lack of the clinical symptoms of inflammation of the mammary gland and the significantly higher level of SCC, PMN, MAC, LYM, ECM, or BII in milk samples. On each plot, the maximal value of each milk indicator for the NSM group (max NSM) was indicated and marked by a dashed line. Numerical data were reported on the box plots using minimum and maximum values, lower and upper quartiles, as well as the median.

Thirdly, fourteen samples of the NCM group were determined based on the results of the first comparison into data series of the NSM group and data series of the SM group. When the SM data series were divided with the NSM data series on the timeline, two independent SM groups were created. On each plot, the NCM subgroups were separated by dotted lines. Univariate marginal distributions of data series of SCC, PMN, MAC, LYM, ECM, and BII were tested again independently for the NSM group and SM group using a univariate Kolmogorov-Smirnov test.

Fourthly, NSM and SM groups were compared with the CM group using the Kruskal-Wallis test followed by Dunn’s multiple comparisons test due to the non-Gaussian distribution of at least one data series in each data being compared. On each plot, the CM group was separated by a solid line. Numerical data were reported on the box plots using minimum and maximum values, lower and upper quartiles, as well as the median.

Finally, the accuracy of subclinical and clinical mastitis detection based on the SCC and ECM was calculated using four thresholds (NSM mean, NSM mean + SD, NSM mean + 2SD, Max NSM), respectively. The sample was annotated as representing mastitis (1) when the individual feature value was above threshold and annotated as representing non-mastitis (0) when below it. The same annotation was done in NSM, SM I, SM II, and CM groups. The sensitivity (Se), specificity (Sp), positive predictive value (PPV), and negative predictive value (NPV) of subclinical mastitis detection were estimated. The values of Se, Sp, PPV, and NPV were calculated across the range 0.1 to 1.0 using standard formulae [43].

## 3. Results

**Figure 2:**
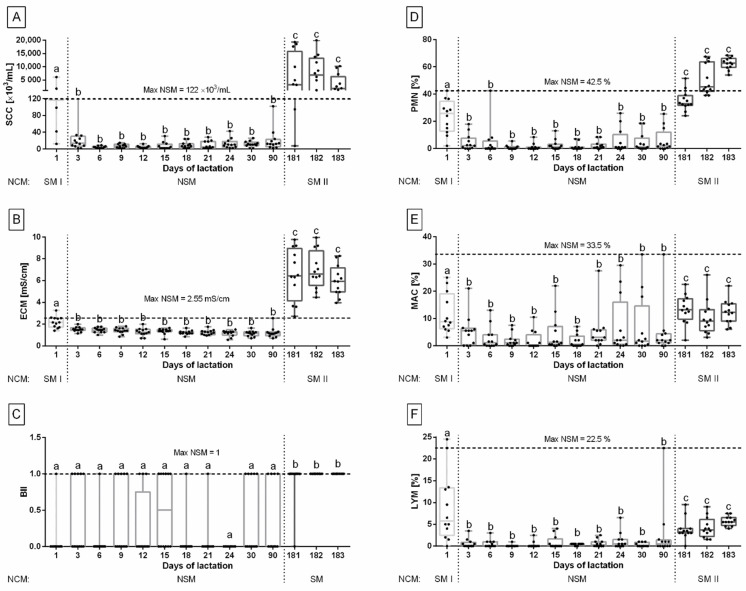
Somatic cell count (SCC) (**A**), electrical conductivity of milk (ECM) (**B**), Bacterial Infection Index (BII) (**C**), percentage of polymorphonuclear neutrophils (PMN) (**D**), percentage of macrophages (MAC) (**E**), and percentage of lymphocytes (LYM) (**F**) (minimum value, lower quartile, median, upper quartile, and maximum values) in the group with no clinical symptoms of mastitis (NCM) in the following days of milk collection (1, 3, 6, 9, 12, 15, 18, 21, 24, 30, 90, 181, 182, 183). The subgroup with no subclinical symptoms of mastitis (NSM) and one (**C**) or two (**A**,**B**,**D**–**F**) subgroups with subclinical symptoms of mastitis (SM, SM I, SM II) were separated by dotted lines. The maximal value of each milk indicator for the NSM group (max NSM) was indicated and marked by a dashed line. Lower case letters (a–c) indicate differences between groups for *p* < 0.05.

Within the NCM group, the value of SCC in the milk differed between the days of sampling (*p <* 0.0001). On day 1 after birth, the value of SCC in the milk was 896.70 ± 449.00 × 10^3^/mL. On day 3 after birth, a decrease in the value of SCC in milk, to the average value of 9.88 ± 10.23 × 10^3^/mL was observed, which persisted until day 90 after birth with no differences between days 3 and 90 after birth. After the weaning (day 181 after birth), an increase in the value of SCC was observed, to the average value of 5995.00 ± 974.70 × 10^3^/mL, which persisted until day 183 after birth with no differences between days 181 and 183 after birth. Based on a lack of differences within consecutive days, the data were grouped into three subgroups represented as follows: (i) day 1 after birth (SM I), (ii) days 3 to 90 after birth (NSM), and (iii) days 181 to 183 after birth (SM II). The max NSM of SCC was 122.00 × 10^3^/mL (Figure 2A).

Within the NCM group, the value of ECM in the milk differed between the days of sampling (*p* < 0.0001). On day 1 after birth, the value of ECM in the milk was 2.15 ± 0.15 mS/cm. On day 3 after birth, a decrease in the ECM, 1.15 ± 0.06 mS/cm, to the average value of 1.2 ± 0.30 mS/cm was observed, which persisted until day 90 after birth with no differences between days 3 and 90. After the weaning, an increase in the value of ECM was observed, to the average value of 6.52 ± 0.29 mS/cm, which persisted until day 183 after birth with no differences between days 181 and 183. Based on a lack of differences within consecutive days, the data were grouped into three subgroups represented as follows: (i) day 1 after birth (SM I), (ii) days 3 to 90 after birth (NSM), and (iii) days 181 to 183 after birth (SM II). The max NSM of ECM was 2.55 mS/cm (Figure 2B).

Within the NCM group, the BII in the milk differed between the days of sampling (*p* < 0.0001). On day 1 after birth, the BII in the milk was 0.08 ± 0.29 indicating that the growth of Coagulase Negative Staphylococci (CNS) was identified in milk from one mare. There were no differences in BII between days 1 and 90 after birth and the average BII in this period was 0.24 ± 0.38. Until day 90 after birth, within 132 milk samples, a growth of CNS was identified in fifteen milk samples, growth of *Rhodococcus equi* was identified in five milk samples, growth of *Streptococcus* sp. was identified in two milk samples, and growth of *Staphylococcus aureus* was identified in one milk sample. Moreover, in another eight milk samples, mixed growth was noted; CNS and *Streptococcus* sp. in four milk samples, *Rhodococcus equi* and *Streptococcus* sp. in five milk samples, *Staphylococcus aureus* and *Streptococcus* sp. in one milk sample, and CNS, *Rhodococcus equi* and *Streptococcus* sp. in one milk sample. After the weaning, an increase in BII was observed, to the average value of 0.97 ± 0.10, which persisted until day 183 after birth with no differences between days 181 and 183. In the weaning period, within 36 milk samples, the growth of CNS was identified in six milk samples, the growth of *Streptococcus equi* subsp. *Zooepidemicus* was identified in nine milk samples, the growth of *Streptococcus dysgalactia* subsp. *equisimils* was identified in three milk samples, a growth of *Rhodococcus equi* was identified in five milk samples, and the growth of other *Streptococcus* sp. was identified in twelve milk samples. Based on a lack of differences within consecutive days, the data were grouped into two subgroups represented as follows: (i) days from 1 to 90 after birth (NSM) and (ii) days 181 to 183 after birth (SM). The max NSM of BII was 1.00 (Figure 2C).

Within the NCM group, the percentage of PMN in the milk differed between the days of sampling (*p <* 0.0001). On day 1 after birth, the percentage of PMN in the milk was 23.46 ± 3.28%. On day 3 after birth, a decrease in the percentage of PMN in the milk, 4.19 ± 1.62%, to the average value of 3.21 ± 0.55% was observed, which persisted until day 90 after birth with no differences between days 3 and 90. After the weaning (day 181 after birth), an increase in the percentage of PMN was observed, to the average value of 48.36 ± 2.30%, which persisted until day 183 after birth with no differences between days 181 and 183. Based on a lack of differences within consecutive days, the data were grouped into three subgroups represented as follows: (i) day 1 after birth (SM I), (ii) days 3 to 90 (NSM), and (iii) days 181 to 183 (SM II). The max NSM of PMN was 42.50% (Figure 2D).

Within the NCM group, the percentage of MAC in the milk differed between the days of sampling (*p* = 0.0004). On day 1 after birth, the percentage of MAC in the milk was 11.96 ± 1.98%. On day 3 after birth, a decrease in the percentage of MAC in milk, 4.96 ± 1.59%, to the average value of 4.23 ± 0.67% was observed, which persisted until day 90 after birth with no differences between days 3 and 90. After the weaning, an increase in the percentage of MAC was observed, to the average value of 11.92 ± 0.87%, which persisted until day 183 after birth with no differences between days 181 and 183. Based on a lack of differences within consecutive days, the data were grouped into three subgroups represented as follows: (i) day 1 after birth (SM I), (ii) days 3 to 90 (NSM), and (iii) days 181 to 183 (SM II). The max NSM of MAC was 33.50% (Figure 2E).

Within the NCM group, the percentage of LYM in the milk differed between the days of sampling (*p* < 0.0001). On day 1 after birth, the percentage of LYM in the milk was 8.69 ± 2.14%. On day 3 after birth, a decrease in the percentage of LYM in milk, 0.61 ± 0.27%, to the average value of 0.53 ± 0.01% was observed, which persisted until day 90 after birth with no differences between days 3 and 90. After the weaning, an increase in the percentage of LYM was observed, to the average value of 4.55 ± 0.34%, which persisted until day 183 after birth with no differences between days 181 and 183. Based on a lack of differences within consecutive days, the data were grouped into three subgroups represented as follows: (i) day 1 after birth (SM I), (ii) days from 3 to 90 (NSM), and (iii) days from 181 to 183 (SM II). The max NSM of LYM was 22.50% (Figure 2F).

**Figure 3:**
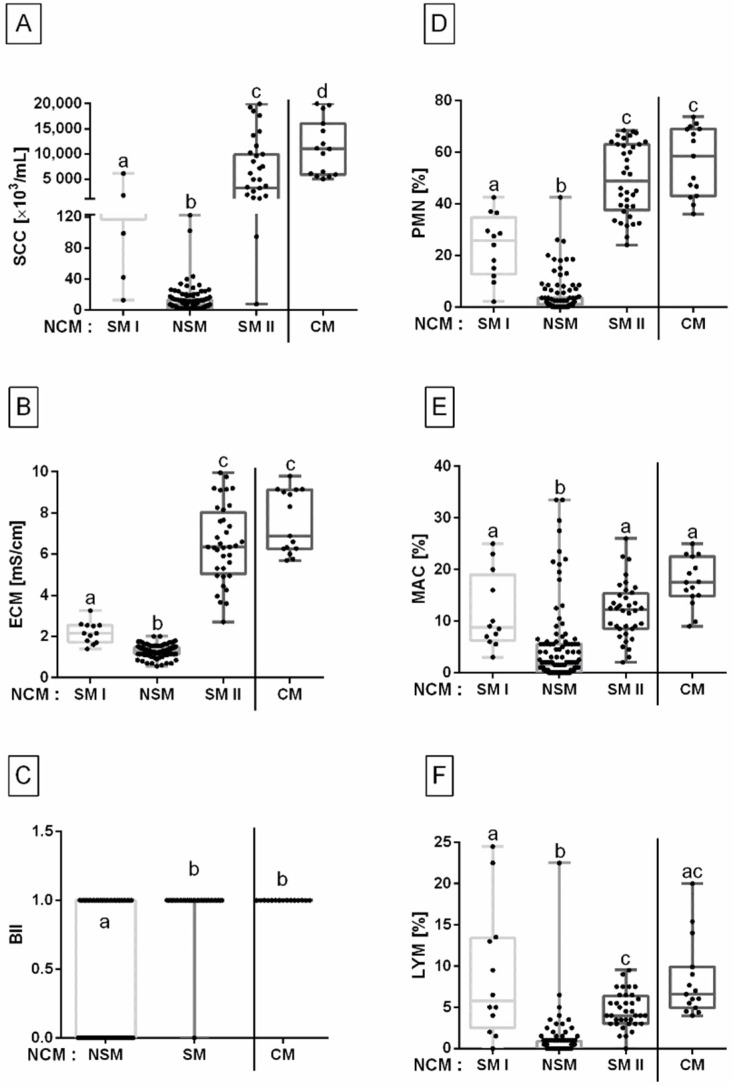
Somatic cell count (SCC) (**A**), electrical conductivity of milk (ECM) (**B**), Bacterial Infection Index (BII) (**C**), percentage of polymorphonuclear neutrophils (PMN) (**D**), percentage of macrophages (MAC) (**E**), and percentage of lymphocytes (LYM) (**F**) (minimum value, lower quartile, median, upper quartile, and maximum values) in the group with clinical symptoms of mastitis (CM) compared to the group with no clinical symptoms of mastitis (NCM), separated into subgroups with no subclinical symptoms of mastitis (NSM) and one (**C**) or two (**A**,**B**,**D**–**F**) subgroups with subclinical symptoms of mastitis (SM, SM I, SM II). The CM group was separated by a solid line. Lower case letters (a–d) indicate differences between groups for *p* < 0.05.

When compared to the CM group, where the value of SCC was 12472.12 ± 2357.33 × 10^3^/mL, a higher value of SCC in CM than in other groups was noted. The value of SCC was higher in SM II than in SM I and NSM, as well as in SM I than in NSM (*p <* 0.0001) (Figure 3A).

In the CM group, the value of ECM was 7.40 ± 0.57 mS/cm. A higher value of ECM in CM than in NSM was noted. No difference was found between CM and SM II, whereas the value of ECM was higher in SM I than in NSM (*p* < 0.0001) (Figure 3B).

In the CM group, the value of BII was 1.00 ± 0.00. Higher BII in CM than in NSM was noted. No difference was found between CM and SM, whereas BII was higher in SM than in NSM (*p* < 0.0001). In the CM group within 15 milk samples, the growth of *CNS* was identified in four milk samples, the growth of *Streptococcus equi* subsp. *zooepidemicus* was identified in three milk samples, the growth of *Rhodococcus equi* was identified in three milk samples, the growth of *Klebsiella pneumonie* was identified in two milk samples, the growth of *Staphylococcus aureus* was identified in one milk sample, and the growth of other *Streptococcus* sp. was identified in two milk samples (Figure 3C).

In the CM group, the percentage of PMN was 57.13 ± 5.10%. A higher percentage of PMN in CM than in SM I and NSM were noted. No difference was found between CM and SM II, whereas the percentage of PMN was higher in SM I than in NSM (*p* < 0.0001) (Figure 3D).

In the CM group, the percentage of MAC was 17.75 ± 1.95%. A higher percentage of MAC in CM than in NSM was noted. No difference was found between CM, SM I, and SM II, whereas the percentage of MAC was higher in SM I and SM II than in NSM (*p <* 0.0002) (Figure 3E).

In the CM group, the percentage of LYM was 8.75 ± 1.96%. A higher percentage of LYM in CM than in NSM was noted. No difference was found between CM, SM I, and SM II, whereas the percentage of LYM was higher in SM I and SM II than in NSM (*p <* 0.0001) (Figure 3F).

For the SCC and ECM in the equine milk, the accuracy of mastitis detection is summarized in Table 1. A salient observation is that the Se decreased with higher threshold values (max NSM < m + 2SD < m + SD < NSM mean) for the SCC in SM I and SM II as well as the ECM in SM I, whereas for the SCC in CM, as well the ECM in SM II and CM, the Se value was always 1.00. Similarly, the NPV decreased with higher threshold values for the same indicators and in the same groups like the Se. The Sp increased with higher threshold values (max NSM > m + 2SD > m + SD > NSM mean) to the maximal 1.00 level for the maximal threshold value, for both evaluated indicators in all mastitis groups. Similarly, the PPV increased with higher threshold values for the same indicators and in the same groups like the Sp, however, for the PPVs, the lowest threshold (NSM mean) was very low (for the SCC in SM I: 0.21; SM II: 0.44; and CM: 0.14 groups; for the ECM in SM I: 0.14; SM II: 0.34; and CM: 0.09 groups).

## 4. Discussion

In mares, the mammary gland is still studied less than other livestock species. To the best of our knowledge, the study presented here for the first time provides the values of indicators measured in equine milk as criteria for subclinical mastitis classification. We hope the obtained results presented on the background of normal lactation and clinical mastitis may be used in the future to design the early detectors of equine mammary gland inflammation and to improve the equine milk quality sourced from dairy equine farms.

The mare’s mammary gland consists of one pair of mammae each with a single teat stocked with two or three teat canals [27]. Each half comprises a single mammary complex, which each has two or three mammary units [26]. Each of the mammary units is a separate unit and contains its milk ducts, the milk sinus, and the teat canal ending with a papillose vent [44]. The equine mammary gland is smaller than the bovine mammary gland and is located in the inguinal area, therefore it is less subjected to trauma and infection [26] however, the first clinical symptoms of mastitis are hard to observe [34]. This specific anatomical and topographic structure may partially explain the limited recognition of subclinical mastitis and the difficult diagnosis and intramammary administration of drugs in a clinical form [26,27,33,34]. It could be also one of the reasons why mastitis is rarely observed on dairy equine farms [23,24,25].

In mares, the development of mastitis usually occurs by an ascending route, through the teat canal [27]. An open teat canal forms a portal of bacterial infection for the mammary gland [35]. Therefore, the natural barrier of the mammary gland against the contamination includes the work of sphincter muscle [44], presence of the keratin plug with bacteriostatic activities [45], and action of cationic proteins [44] within the teat canal. When a damaging factor, such as a pathogen breaks the natural barrier of the mammary gland, the second line of defense is initiated in the form of leukocyte infiltration, in particular polymorphonuclear neutrophils (PMN), macrophages (MAC), and lymphocytes (LYM) [27,34]. During inflammation, the SCC in milk consists of 99% leukocytes, whereas the remaining 1% are secretory epithelial cells. This cell migration causes a significant increase in the SCC as the total number of cells per mL in milk [27,46] and it can be successfully used in the diagnosis of clinical mastitis in mares [34] and monitoring of the mammary gland health status on dairy equine farms [23,24,25]. In this study, we hypothesized that SCC can also possibly be used to diagnose subclinical mastitis. In this study, it is easy to see that during normal lactation (NCM group) all indicators measured in milk increased in two periods—on the first day after birth, and during the weaning period. Based on the subclinical mastitis criteria [26,28], those two periods have been separated as SM I and SM II, which may indicate the physiological development or a predisposition to developing subclinical mastitis in those periods of lactation [30]. In SM II, the level of SCC, PMN, MAC, LYM, ECM, and BII increased to the level represented by the CM. On the other hand, in SM I, only the level of MAC and LYM achieved comparable results. However, the level of SCC, PMN, and ECM in SM I was higher than in the NSM group. Although there are presently no exact criteria or standards for equine milk classification, one might conclude the recognition of subclinical mastitis in those cases, as received from a healthy or subclinically inflamed udder [26,27,28,30].

In recent studies, the physiological SCC in equine milk was reported as 194 × 10^3^/mL [41] and 377 × 10^3^/mL [47] on the first day postpartum, as well as 34 × 10^3^/mL [41] and 46 × 10^3^/mL [47] in the following days of normal lactation, respectively. However, both authors did not report SCC in the weaning period or the case of mastitis [41,47]. The results presented here are then in line with the recently described results. The slightly higher SCC received here on the first day postpartum may be a result of the applied techniques of SCC quantification in equine milk samples. Numerous authors presented various SCC values in equine milk during the entire normal lactation presented, which ranged from 17 to 52 × 10^3^/mL [47], 25 × 10^3^/mL [48], 39 × 10^3^/mL [49], to 365 × 10^3^/mL [50]. It may be stated that equine milk demonstrated a high hygienic status [41,47,48,49,50]. When compared to the milk from the other livestock animals, equine milk [39,41,47,48,49,50] demonstrates the lowest SCC compared to cows (100–200 × 10^3^/mL) [51,52,53], sheep (300 × 10^3^/mL), or goats (about 1 million/mL) [19]. However, it should be kept in mind that the SCC in equine milk may be affected by the breed, age, sequence, and the month of lactation [39]. Following Prestes et al. [54], the display of clinical symptoms of mastitis takes place after exceeding 500 × 10^3^/mL SCC. On the other hand, Böhm et al. [34] set this limit at 100 × 10^3^/mL during lactation and 400 × 10^3^/mL after weaning. In this study, the transition from subclinical mastitis (5995 × 10^3^/mL) to clinical (12472 × 10^3^/mL) form was noted in the higher SCC values. It suggests that the mares’ udder may have a considerable physiological reserve limiting the demonstration of clinical symptoms of inflammation [30,43]. On the other hand, Motta et al. [35] reported 247.57 × 10^3^/mL SCC in equine milk without the presence of pathogens and 1621.86 × 10^3^/mL SCC in microbiologically infected equine milk. In this study, BII was high both in SM II and CM groups and therefore in line with recent reports. In SM II, the residual milk in the gland can exert pressure on the teat canal, the first natural and mechanical barrier against the contamination of the mammary gland [43] leading to the opening of the teat canal and thus opening a portal of bacterial infection to the mammary gland [35]. After weaning, accumulated milk may potentially drip through the teat canal, facilitating the entrance of infectious agents [55], compromising the anatomical barrier of the teat canal [27]. It should be kept in mind, more often half of the udder is infected via the ascending, also referred to as galactogenic route, through the teat canal [27,55]. In SM I, when the increase of SCC with no increase of BII was noted, other mechanisms of a natural predisposition to developing subclinical mastitis should be taken into account [30]. In this period, the changes in the permeability of the blood-mammary gland barrier [54] and/or the effect of the secretion of colostrum without the active inflammatory process [41,46] may be suspected, since conditions of the milk residual and the anatomical barrier compromise have not been met [27], which required further research.

In this study, the clinical symptoms occurred in mares in 40.0% of the CM group after foaling (both natural and via cesarean section) and in 26.7% after weaning foals, which is in line with both the supposed predisposing periods to developing subclinical mastitis and with recent studies. Recently, the clinical form of mastitis has most often been reported in mares after giving birth and after weaning foals [26,33,34,56]. Moreover, in this study, *Streptococcus equi* subsp. *zooepidemicus*, *Rhodococcus equi, CNS*, *Streptococcus* ssp, *Staphylococcus aureus*, and *Klebsiella pneumonie* were pathogens most commonly isolated from equine milk both in SM after weaning and in CM groups, similar to recent research [26,30,33,34,56]. Those pathogens were found on the skin of the udder and have been isolated from the milk of healthy post-partum mares [34,57]. One might suggest a link between the clinical outcome of mastitis and the breakdown in the innate immune response or disturbance between the host and microbe [26], especially when the teat canal barrier is compromised [27]. Moreover, the differences in the content of immune cells in SM I and SM II have additionally supported the above hypothesis on predisposition to developing subclinical mastitis. As PMN is one of the most important defense mechanisms that cleans the mammary gland from pathogens [30,35], their preponderance in SM II when BII was increased over SM I when BII was not increased, is completely justified. Likewise, the preponderance of LYM in SM I over SM II, one of the most important parts of acquired immunity both in the tissue fluids and the blood, again indicates the permeability of the blood-mammary gland barrier [55] as a direction of further research in the early postpartum period. Those findings prove early postpartum and weaning periods as particularly sensitive and prone to the development of udder inflammation in mares.

During inflammation of the mammary gland, an imbalance in the permeability of the capillaries occurs. The endothelium of the capillaries and the intercellular gap junctions, as well as the ionic pump system in the cell wall become damaged, which causes changes in the ionic composition of the milk. Sodium (Na^+^) and Chlorine (Cl^−^) ions enter into the extracellular space, and thus into the lumen of the mammary gland and to the milk, through the apical part of the epithelial cells of the gland [36,58]. Moreover, under inflammatory conditions in the mammary gland, the concentration of lactose in milk decreases, and Na^+^ and Cl^−^ pass from blood to milk in order to maintain an adequate osmotic pressure [28,58] as lactose is considered to be the main determinant of osmosity in milk [28,59]. Therefore, in dairy farmed animals, the ECM, which is a measure of the resistance of the given material to the action of electric current [31], is used as the commercial indicator of mastitis [6,60]. In mares, the ECM has recently been successfully evaluated in normal equine milk [41,42,49], however, the ECM in the case of subclinical and clinical equine mastitis has not been investigated yet. In recent studies, the physiological ECM in equine milk was reported as ranging from 1.9 to 4.6 mS/cm in normal lactation [41,49]. Again, both authors did not report ECM in the weaning period or the case of mastitis [48,49]. The results presented here in NSM are in line with the recently described results, however, both in SM II and CM those results are higher. One might observe that this study fills the gap in the available literature on changes in ECM of equine milk during the weaning period and in the case of mammary gland inflammation. It is worth noting that an increase in ECM, SCC, and PMN co-occurred in the SM I, SM II, and CM groups. As in SM I, the effect of the secretion of colostrum without the active inflammatory process [41,46] should be considered, the co-occurred in SM II and CM groups can be carefully considered as an early symptom of subclinical mastitis. However, this preliminary study indicates some commonalities of the measured indicators of equine milk, their application as the indicators in early detection of mastitis, or the tool of assessment of the health status of the mammary gland in dairy equine farms requires further research.

The decrease of the Se values refers to the SCC’s and ECM’s ability to detect the mastitis when the mastitis is present [43,61] in selected groups caused by more and more false-negative samples being below a threshold. In the case of the CM group, for both evaluated indicators, the samples values were much higher than each of the considered thresholds. Similarly, the NPV refers to the probability that a negative SCC’s and ECM’s result correctly predicts the absence of mastitis [43,61] which decreased in the corresponding groups. These results are in agreement with the relationship between SCC and subclinical mastitis in lactating dairy cows, where Se increased when the threshold level of SCC was lowered [62]. The increase of the Sp values referring to the SCC’s and ECM’s ability to exclude the mastitis when the mastitis is absent [43,61], is justified as well as having the highest value when a threshold is the max NSM since none of the samples in the NSM group can be classified as false-positive. Similarly, the PPV, which refers to the probability that a positive SCC’s and ECM’s result correctly predicts the presence of mastitis [43,61], increased in the corresponding groups. These results are in also agree with the recent research in lactating dairy cows, where Sp increased when the threshold was raised [62]. The cow-level SCC threshold of 100 × 10^3^ cells/mL was considered appropriate to identify subclinical mastitis of lactating dairy cows concerning the results in less false-negative outcomes [62]. However, the current results are not enough to suggest similar conclusions. The obtained results indicate the necessity to conduct further studies on the correct selection of thresholds for the SCC and ECM evaluation in equine mild, on a larger group of mares and using additional data analysis, as the tradeoff between the Se and Sp is explored in ROC analysis [43]. However, based on the current preliminary results, this direction of further research seems to be promising, especially since the accuracy of mastitis detection based on the SCC and ECM in milk has not been investigated in any of the recent research on equine mastitis [23,24,25,26,30,32,33,34,35,36,39,40,41,42,47,48,49,50,54,56,57]. Therefore, only as a reference, the accuracy of mastitis detection in lactating dairy cows is reported here. For the 100 × 10^3^ cells/mL threshold of SCC, the Se, Sp, PPV, and NPV of subclinical mastitis detection ranged from 0.53, 0.96, 0.87, and 0.79 to 0.61, 0.96, 0.80, and 0.90, respectively, depending on the presence and type of the pathogens [62]. The Se and Sp of ECM-based clinical mastitis detection based ranged from 0.16 and 0.92 to 0.48 and 0.98, respectively; whereas subclinical one from 0.03 and 0.91 to 0.19 and 0.98, respectively, depending on EC traits and division to subsets [63].

## 5. Conclusions

An increase in the level of examined indicators in equine milk, especially SCC and ECM, may be an early symptom of subclinical mastitis. In the weaning time, the physiological development or a predisposition to developing subclinical mastitis should be considered. Further research is required to determine the usefulness of SCC and ECM measurements in equine milk as indicators in the early detection of mammary gland inflammation.

## Figures and Tables

**Table 1:** The sensitivity (Se), specificity (Sp), positive predictive value (PPV), and negative predictive value (NPV) of mastitis detection in the first subgroup with subclinical symptoms of mastitis (SM I), the second subgroup with subclinical symptoms of mastitis (SM II), and the group with clinical symptoms of mastitis (CM) based on the values of the somatic cell count (SCC) and electrical conductivity of milk (ECM) in the equine milk. The Se, Sp, PPV, and NPV were estimated based on four thresholds representing the group with no subclinical symptoms of mastitis (NSM); NSM mean; NSM mean + SD (m + SD); NSM mean + 2SD (m + 2SD); the maximal value of each milk indicator for the NSM group (max NSM)).

Groups	SM I				SM II				CM			
Threshold	NSM Mean	m + SD	m + 2SD	Max NSM	NSM Mean	m + SD	m + 2SD	Max NSM	NSM Mean	m + SD	m + 2SD	Max NSM
SCC in the milk												
Se	1.00	0.92	0.92	0.77	0.97	0.97	0.97	0.95	1.00	1.00	1.00	1.00
Sp	0.63	0.88	0.95	1.00	0.63	0.88	0.95	1.00	0.63	0.88	0.95	1.00
PPV	0.21	0.43	0.63	1.00	0.44	0.70	0.84	1.00	0.14	0.33	0.53	1.00
NPV	1.00	0.99	0.99	0.98	0.99	0.99	0.99	0.98	1.00	1.00	1.00	1.00
ECM in the milk												
Se	1.00	0.92	0.62	0.15	1.00	1.00	1.00	1.00	1.00	1.00	1.00	1.00
Sp	0.41	0.82	0.98	1.00	0.41	0.82	0.98	1.00	0.41	0.82	0.98	1.00
PPV	0.14	0.34	0.73	1.00	0.34	0.63	0.93	1.00	0.09	0.26	0.73	1.00
NPV	1.00	0.99	0.96	0.92	1.00	1.00	1.00	1.00	1.00	1.00	1.00	1.00

## Data Availability

The data presented in this study are available on request from the corresponding author.
